# Scanning X-ray microdiffraction studies of protein accumulation and tissue loss in thin sections of human brain tissue in Alzheimer’s disease

**DOI:** 10.21203/rs.3.rs-9679677/v1

**Published:** 2026-05-31

**Authors:** Prakash Nepal, Abdullah Al Bashit, Theresa Connors Stewart, Derek H. Oakley, J. Zwang Theodore, Bradley T. Hyman, Lee Makowski

**Affiliations:** Northeastern University; Northeastern University; Massachusetts General Hospital; Harvard University; Massachusetts General Hospital; Massachusetts General Hospital; Northeastern University

## Abstract

Neurodegeneration in Alzheimer’s disease is characterized by accumulation of pathological protein deposits including Aβ plaques, tau tangles and diffuse neuropil threads accompanied by wide-spread tissue loss. While dense protein deposits are readily observed by conventional methods, histology struggles to properly capture microscopic evidence of absences corresponding to tissue loss. This study demonstrates the use of scanning X-ray microdiffraction to quantitatively evaluate both protein accumulation and tissue loss in thin sections of human brain tissue across anatomical regions and stages of disease. We show that scanning small-angle microdiffraction can quantify the material properties of tissue through mapping the presence of sub-micron-sized “scattering voids”, imaging a tissue attribute that is typically invisible and providing an alternative metric for tissue integrity. Correlation of x-ray data with images of silver-stained sections provided quantitative assessment of protein accumulation readily observed in densely stained features. Regions containing large and abundant scattering voids proved to be highly fragile and closely correspond to locations that were damaged during subsequent tissue processing. This work demonstrates the use of scanning microdiffraction for mapping the abundance of these voids in tissue, providing a detailed description of a phenomena that is typically invisible to histology and utilizing it to study tissue structure across multiple brain regions in diseased human tissue.

## Introduction

Alzheimer’s disease is a progressive neurodegenerative disease in which pathological processes slowly spread through the brain leading to neuronal death and consequent cognitive decline. During disease progression, neurodegeneration is accompanied by the formation of Aβ plaques (Cohen et al., 2015; Di Fede et al., 2018) and tau-containing neurofibrillary tangles (Shi et al., 2021) that were first observed by Alois Alzheimer and are readily observed by standard techniques. In addition to these pathological hallmarks, there is widespread deposition of tau as neuropil threads and other diffuse structures (Dickson, 1997) and pockets of tissue thinning that corresponds to gross tissue loss. These attributes are far more difficult to detect or quantitate using conventional methods. Here we introduce scanning x-ray microdiffraction as an alternative approach to quantify these features.

The profound heterogeneity of human brain tissue makes the use of x-ray scattering for its study challenging. However, the use of micro- and nano-beams at synchrotron x-ray sources has recently enabled measurement of scattering from small volumes (e.g. ~250 μm^3^), including use with thinsections of brain tissue (Liu and Makowski, 2023; Nepal et al., 2024). The pathological cross-β fibrils characteristic of protein deposits associated with Alzheimer’s and other neurogenerative diseases (Chen et. al. 2017) are highly resilient to the chemical and physical processes required to prepare fixed, sectioned tissue (Liu et al., 2016; Zohdi et al., 2015; Werner et al., 2000). X-ray scattering from cross-β fibrils exhibits a characteristic scattering ‘fingerprint’ that includes a strong peak at a scattering angle corresponding to a spacing of 4.7 Å (the distance between β-strands in the fibrils) and a second, somewhat weaker peak, at 10 Å spacing (the distance between β-sheets in the fibrils). Diffuse deposits in which the principal molecular constituents are not well ordered are more difficult to detect (Liu et al., 2025). Although they give rise to scattering at the same scattering angles as the cross-β fibrils, the scattering is weaker and difficult to differentiate from scattering of healthy tissue.

We have demonstrated that small-angle scattering (SAXS) from tissue provides additional insight into its molecular organization (Nepal et al., 2024). SAXS from human brain tissue exhibits a characteristic linear dependence of log(I) as a function of log(q) where I is the scattered intensity and q is roughly proportional to scattering angle (strictly, it is the momentum transfer defined as q = 4π sin(q)/l, where 2q is the scattering angle and l is the wavelength of the incident x-rays). Generally, a linear relationship of log(I) as a function of log(q) has been interpreted as indicating the presence of fractal-like structures (Martin & Hurd, 1987; Suzuki et al., 1997). However, there is no evidence to support the notion that fixed human brain tissue has a fractal-like structural organization. The origin of this scale-free behavior of small-angle scattering became clear only after we discovered that the intensity of small-angle scattering from these tissue samples has no correlation with the intensity at wide angles (WAXS), indicating that the structures responsible for the SAXS intensity are distinct from those that scatter at wide angles. Furthermore, the scattering exponent (negative of the slope of the log-log curve at small angles) is **inversely** proportional to the scattered intensity at wide-angles. We interpreted this as indicating that the major contributor of SAXS intensity was sub-micron sized scattering voids in the tissue (Nepal et al., 2024). The large electron density difference between voids and tissue leads to intense scattering (Nepal & Saldin, 2018) dominating the small-angle region of these patterns. Furthermore, we demonstrated that a scale-free distribution of void sizes would give rise to a linear log-log scattering intensity at small angles, thereby explaining the linear form without the need to invoke the presence of fractal-like structures. In this case, a scale-free distribution of void sizes indicates that the log of the abundance of voids is inversely proportional to the log of their sizes (i.e., smaller voids are far more abundant than larger ones). The voids – literally tiny holes in the dried, fixed tissue - apparently form during tissue processing that involves formalin cross-linking and dehydration. Our data indicate that most voids formed during tissue processing are under 0.1 μ in diameter, but the possible presence of larger, less abundant voids cannot be ruled out.

We analyze the size and abundance of these voids in tissue thin sections on three length scales. In the scattering volume from a single micro-diffraction exposure (~ 5×5×20 μ^3^) we calculate the ‘scattering exponent’, p, from the slope of the log(I) vs log(q) curve (i.e., I ~ q^p^) at small scattering angles (Nepal et al., 2024). Modeling that scattering as due to a scale-free distribution of void sizes (i.e., assuming that the abundance, N, of voids as a function of radius is distributed in a power-law form, N(r) ~ r^−a^, where ‘a’ is the radial exponent), we can use the scattering exponent, p, to calculate the average radius of voids within that single scattering volume (Nepal et al., 2024). This provides a measure of the porosity of the tissue irradiated in a single x-ray exposure. The voids are ubiquitous in this material - present throughout the tissue. Most are much smaller than 0.1 μ in radius and their characterization provides a quantitative measure of the material properties of these histologically prepared sections, one pixel at a time. On a larger length scale, scanning x-ray microdiffraction was used to map variation of these material properties across a thin section. Mapping the variation of scattering exponent over a 300×300 μ^2^ region of interest (ROI), we often observe local pockets of unusually high scattering exponent, indicative of a high abundance of voids and suggesting a relatively diaphanous structure compared to the surrounding tissue. Finally, we observed significant variation in the *average* scattering exponent of different ROIs, suggesting that, on the length scale of millimeters, there may be variations in the texture of macromolecular structure within brain tissue.

Here, we supplement our x-ray data by comparison with images of the samples that were silver stained after x-ray exposure. Silver-staining is a widely used method for visualizing subcellular structures in histological thin sections (Switzer et.al. 1979; Grizzle et. al. 1996; Uchihara 2007). Exposure of tissue to scanning x-ray microdiffraction alters the surface charge of the tissue by ejecting electrons, thereby lowering its affinity for silver stain. As we will demonstrate, regions of interest (ROIs) that are silver-stained after exposure to x-rays are readily visualized, making possible accurate registration of images of silver-stained tissue with heat maps generated from x-ray data.

Silver-staining of tissue makes possible visualization of diffuse protein deposits the density of which may vary widely even within a single tissue section (Rosenwald et.al. 1993; Gallyas 2008). Comparison of the apparent density of these deposits with the SAXS scattering exponent revealed an inverse correlation of the density of protein deposits with size and abundance of sub-micron scattering voids. It seems intuitively clear that dense protein deposits might suppress the formation of voids during tissue processing and our data supports this supposition.

Here we utilize scanning x-ray microdiffraction of fixed human brain tissue in coordination with microscopy of silver-stained tissue to map the density of macromolecular deposits across six regions of the brains of Alzheimer’s disease subjects exhibiting Braak stages II, IV and VI. We show that protein deposition is substantial even at early stages of disease and that regions exhibiting larger and more abundant voids have much lower physical resilience during tissue processing and staining.

These methods represent an alternative descriptor of tissue integrity that is not fully captured by conventional histology, but which correlates with histologically-defined features within the tissue. While one of the main readouts of neurodegeneration is tissue loss, it is challenging for histology to properly capture an “absence”. Here we demonstrate that scanning small-angle x-ray diffraction can map the locations of “absences” in which there is a high abundance of scattering voids in tissue allowing a description of this phenomena in a way that is typically invisible to histology. This work is an initial probe to determine the degree to which these scattering results can be used as a readout for such phenomena, providing a novel window into tissue loss and fragility in disease.

## Methods

Human brain tissue was obtained from the Massachusetts Alzheimer’s Disease Research Center Brain Bank. All subjects or their authorized next of kin provided written informed consent for brain donation and the use of tissue for research purposes in accordance with institutional guidelines. Clinical and neuropathological evaluations were performed according to standardized criteria established by the Center. The study protocol, including tissue procurement and experimental procedures, was reviewed and approved by the Massachusetts General Hospital Institutional Review Board and conducted in compliance with all relevant ethical regulations.

Tissue samples were prepared as previously described using standard neuropathological protocols (Liu et al., 2016). Sections were cut to a thickness of 20 μm—thicker than conventional histological sections—to increase the irradiated volume during X-ray microdiffraction (XMD). These unstained sections exhibit chemical and physical properties characteristic of histological tissue preparations. Serial sections stained for Aβ and tau pathology were also prepared in order to identify regions of interest (ROIs) for x-ray scanning and provide positive identification of the principal constituent of lesions. Tissue sections were mounted on 12 μm thick, 1 cm × 1 cm mica films and affixed to 3D-printed sample holders designed to LiX beamline specifications.

Data collection was conducted at the LiX beamline of the NSLS-II synchrotron source at Brookhaven National Laboratory (Yang et al., 2020; 2022). Sample holders were mounted directly on the LiX stage in a vacuum environment. Each tissue section was scanned using a 5 μm diameter X-ray microbeam on a square grid with 5 μm spacing. Scanned areas ranged from 300 × 300 μm^2^ (yielding 3,600 diffraction patterns) to 600 × 600 μm^2^ (14,400 patterns). Exposure time was 0.5 seconds, but including data transfer and stage movement, each pattern took approximately 0.8 seconds, resulting in a total scan time of about 48 minutes for a 300 × 300 μm^2^ ROI.

Scattering data were simultaneously collected using both SAXS and WAXS detectors, circularly averaged, and merged using LiX-specific software. Data processing included geometric corrections, transmission and thickness normalization, and other standard adjustments based on validated LiX protocols (Yang et al., 2013; 2020). Scattering intensities were estimated at 570 *q*-values over a range of 0.005 ≤ *q* ≤ 2.7 Å^−1^.

Data were collected for 56 ROIs among the 6 brains analyzed, with at least 3600 diffraction patterns per ROI for a total of over 200,000 diffraction patterns. The scattering volume for each exposure is roughly 5×5×20 μm^3^ (where 20 μm is the thickness of the section), or 500 μm^3^, which is too large for examination of subcellular architecture, but adequate for assessing the status of cellular health within a tissue.

To assess radiation damage, multiple scans of identical tissue regions were performed. No significant alterations in fibrillar scattering were observed. Within a single ROI scan, repeated measures also allowed correction for potential intensity scaling artifacts. This was true even in regions that were later silver-stained and shown to be ‘bleached’ by x-ray exposure. Our interpretation of the impact of x-ray exposure on silver staining is that the incident x-rays eject electrons from the tissue surface, lowering the negative charge distribution of the section, thereby lowering it’s affinity for the positively charged silver ions in the stains. Our observations suggest this occurs without significantly altering the macromolecular structure within the section. Within the ROIs scanned by x-rays we observed a periodic oscillation in the density of silver staining. This appears to be generated by variation in the dead time between x-ray exposures in the ROI, that led to a periodic variation in total x-ray exposure to the tissue (without impacting the exposure time used to collect scattering data). Section thickness was occasionally found to vary at tissue edges due to shrinkage. This variation was readily identifiable when present. Preprocessing steps included subtraction of mica scattering background as detailed previously (Bashit et al., 2022).

Quantitation of the number of neurofibrillary tangles in each section was carried out by image segmentation utilizing pixel classifier. High-resolution VS120 images were preprocessed in ImageJ by fourfold downsampling and conversion to TIFF format. The images were analyzed in Ilastik (ilastik: interactive machine learning for (bio) image analysis) using a supervised pixel-classification approach, in which neurofibrillary tangles and background were manually annotated as separate classes on an image-by-image basis (Berg et. al. 2019). The trained model produced pixel-wise probability maps (0–1) indicating tangle likelihood. These maps, together with the original images, were used in Ilastik’s object-classification workflow to group high-probability pixels into discrete objects, which were manually classified as tangle or non-tangle. Object-level results, including counts per image and associated confidence scores, were exported, and final tangle counts were confirmed by visual inspection using a minimum probability threshold of 0.75 across all images (Berg et. al. 2019).

## Results

### 3.1 Scanning x-ray microdiffraction

Data were collected at the LiX beam line at the NSLS-II synchrotron source at Brookhaven National Laboratory. Tissue sections were prepared from the brains of six Alzheimer’s subjects in regions along the Braak-Tau trajectory including the entorhinal cortex (EC), hippocampus (HC), parahippocampal gyrus (PHG), anterior cingulate cortex (CING), associative visual cortex (V2) and primary visual cortex (V1). The six subjects chosen included two each that were staged at Braak II (mild disease), Braak IV (moderate disease) and Braak VI (advanced disease). Data were collected on 1–3 regions of interest (ROI) from each of these brains/regions, totaling 56 ROIs and over 200,000 diffraction patterns.

**Figure 1:**
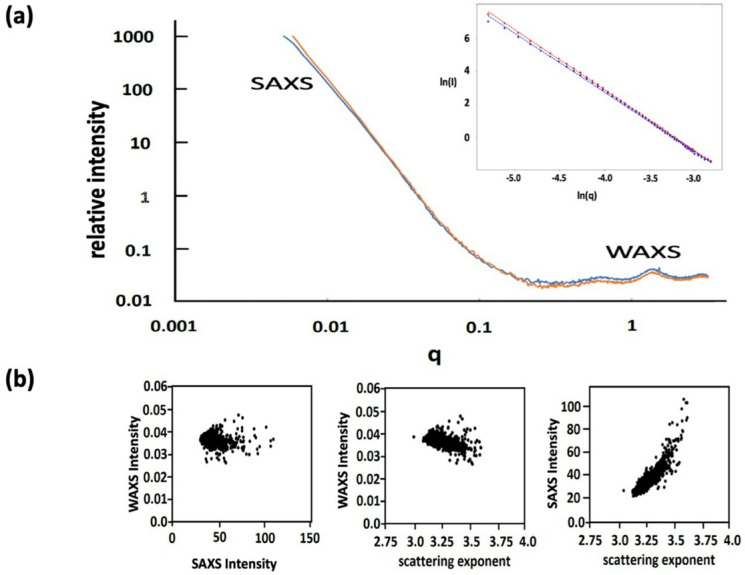
SAXS and WAXS Intensity Distribution **(a)** A log-log plot of scattering intensity, I, versus momentum transfer, q, for two scattering patterns from the same ROI. The intensities in the SAXS and WAXS regimes of these two patterns are inversely correlated (as was commonly observed): the pattern with greater intensity in the SAXS regime exhibited weaker intensity in the WAXS regime. INSET is an enlargement of the smallest angle scattering. **(b)** Scatter plots demonstrating degree of correlation (or lack thereof) among attributes of the observed scattering. Each dot represents a single diffraction pattern within a single ROI. Left Panel: SAXS intensity (at q = 0.01Å^−1^) vs WAXS intensity (at q =1.34 Å^−1^). Middle Panel: WAXS intensity vs scattering exponent, p. Right Panel: SAXS intensity vs scattering exponent, p. Correlation coefficients for the three cases were −0.167, −0.426 and 0.883 respectively.

Figure 1a includes plots of two scattering patterns from the same ROI (EC, Braak VI). We observed that log(I)-log(q) plots from these samples were linear in the range 0.01 ≤ q ≤ 0.1Å^−1^. The patterns in Figure 1a were chosen to demonstrate commonly observed aspects of the scattering including the linearity of the log-log plot at low q and the inverse relationship between intensity in the SAXS and WAXS regimes. It was very common to observe pairs of patterns (such as these) in which the pattern with the higher SAXS intensity exhibited lower WAXS intensity. Figure 1b demonstrates that this is a common feature of scattering from human brain tissue. This is not expected for homogeneous samples and led us to conclude that the constituents responsible for the preponderance of scattering in the SAXS regime were distinct from those responsible for the majority of the WAXS intensity (Nepal et al., 2024).

The slope of the linear portion of the log-log plot at small angles provides a powerful means by which to compare properties of materials prepared in different ways at different times because it is independent of experimental parameters that are difficult to control including sample thickness. Its power-law behavior (Nepal et al., 2024) is often written as an exponential where

$${I\;\alpha \;q}^{-p}$$

and *p*, is the ‘scattering exponent’ which, in general, is non-integer. For the samples of brain tissue we examined, the scattering exponent varied between 3 and 4. This ‘power-law’ behavior has often been interpreted as being due to scattering from fractals (Martin et.al. 1985). However, we were able to show that – in these samples - it is due to scattering from a population of sub-micron sized voids (Nepal et al., 2024), the abundances of which are highly dependent on size and distributed in a power-law form:

$${\text{N}(\text{r})\sim \text{r}}^{-\text{a}}$$

where N is the number of scattering objects with radius r and ‘a’ is the ‘radial exponent’, reflecting the average size of voids within the scattering volume. In general, as size and abundance of scattering particles increases, ‘p’ also increases. As such, the scattering exponent provides a experimentally tractable measure of the size distribution of voids in fixed brain tissue. The inverse correlation of void size to macromolecular density also implies that the scattering exponent provides a quantitative assessment of macromolecular density with larger values of ‘p’ indicating lower tissue density.

An example of the relationship between SAXS and WAXS intensity and scattering exponent is shown in Fig. 1b, which includes scatter plots of small-angle and wide-angle intensities and scattering exponents for the 3721 patterns within a single 61×61 ROI. Left panel of Fig. 1b demonstrates that the intensity of scattering in the small- and wide-angle regimes is uncorrelated (correlation coefficient of −0.167), indicating that they are due to distinct scattering features within the tissue (in this case, voids and macromolecular constituents, respectively). Middle panel of Fig. 1b demonstrates that WAXS intensity is negatively correlated with scattering exponent (correlation coefficient equal to −0.426). This is an immediate consequence of the fact that as the size and abundance of voids increases the volume available for macromolecular constituents that scatter at wide angles decreases. As seen in right panel of Fig. 1b, the SAXS intensities are highly correlated with the scattering exponent (correlation coefficient of 0.883). This is because both are highly sensitive to the size of the scattering objects (in this case voids). Larger voids give rise to higher scattering exponents and stronger small-angle scattering.

### 3.2 Variation of tissue density within individual ROIs

**Figure 2:**
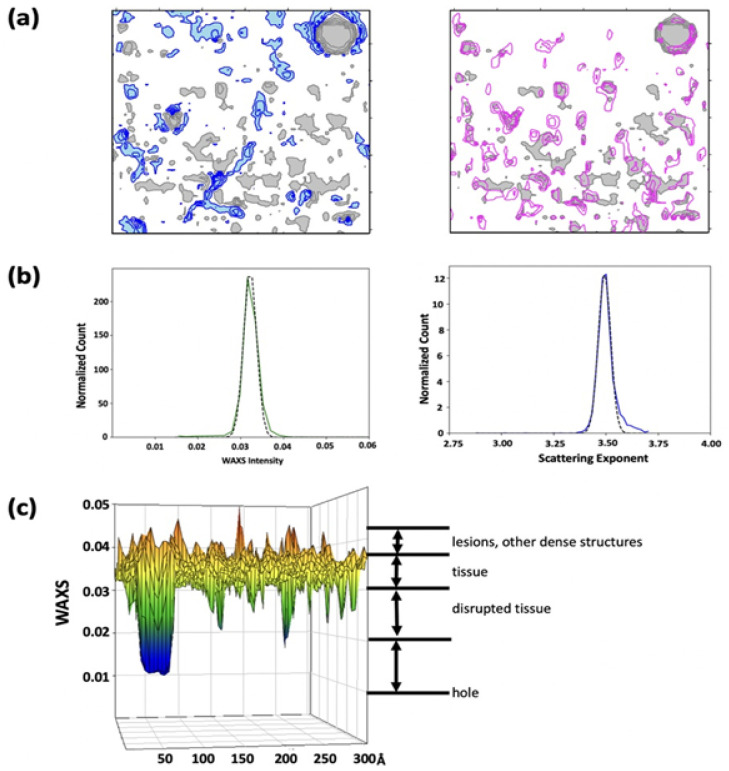
WAXS Intensity and Scattering Exponent Distribution **(a)**Variation of WAXS intensity and scattering exponent across a 300×300 μm^2^ ROI of the entorhinal cortex of a Braak VI case. Left Panel: The regions of greatest WAXS intensity are marked in blue; the lowest WAXS intensity in grey. Regions of high WAXS intensity can usually be identified by their shape as pathological lesions, vascular walls, or other tissue features of relatively high density. Right Panel: Regions of greatest scattering exponent (pink) are frequently found co-located with regions of lowest WAXS intensity. **(b)** Left Panel: Histogram of WAXS intensities in the ROI shown in **2(a)**. Right Panel: Histogram of scattering exponents for the ROI in **2(a)**. **(c)** WAXS intensity distribution in a 300×300 μm^2^ ROI. This is the same data as in **2(a - left)** but replotted to provide a view of the degree to which the intensity varies.

Scanning XMD is an imaging technique in which any attribute of the scattering pattern can be used to create images that show the distribution of that attribute across an ROI. For this report, we will limit our analysis to the intensity of wide-angle scattering (at q = 1.34 Å^−1^ – corresponding to the scattering angle of the cross-β peak) and the scattering exponent (the negative of the slope of the log(I) vs log(q) curve at small angles). Figure 2 **s**hows contour maps of the WAXS intensity and scattering exponent for ROIs collected from a section from the entorhinal cortex of a subject with advanced Alzheimer’s disease (Braak VI).

The map of WAXS intensity is dominated by features that have a high density of macromolecular constituents - for instance, pathological deposits made up of cross-β fibrillar structures such as amyloid plaques or neurofibrillary tangles, or non-pathological features such as vascular walls. Conversely, the most prominent features in the scattering exponent map are pockets of particularly low concentrations of macromolecular constituents, and high abundance of voids. As indicated in the figure, regions with an abundance of voids tend to fall in between the high-density features seen in the WAXS heat maps.

Surprisingly, when a histogram of the WAXS intensities within an ROI is constructed (as in Fig. 2b **Left Panel**), we observe that the distribution of intensities is near-Gaussian in form, deviating from Gaussian only in the presence of a tail extending away from the core of the distribution towards higher intensity. This tail corresponds to the scattering patterns from high-density features and those ROIs with a larger number of high-density features have more prominent high-intensity tails. The inference is that the remainder of the tissue (that occupying the space between the high-density features) has a density (when probed with a 5 μm x-ray beam) that is randomly distributed. This may be due to the averaging intrinsic to probing the complexity of cellular architecture with a 5 μm beam. We would hypothesize that a similar experiment with 1μm beam or smaller would exhibit a broader distribution of intensities.

Similarly, histograms of the scattering exponent (e.g., Fig. 2b **Right Panel**) within an ROI also exhibit near-Gaussian shape with the greatest deviation towards larger values of the exponent. This large-exponent tail corresponds to regions in the ROI with the greatest abundance of large voids. When the distribution of scattering exponent is compared to the corresponding distribution of WAXS intensity we find that the two maps have peaks in regions that rarely overlap – as seen from a comparison of Figs. 2a.

Figure 2c is an alternative visualization of the distribution of WAXS intensity within the same ROI as Fig. 2a and 2b, displayed here as a three-dimensional plot. A hole in the section, caused by the intersection of a small blood vessel, provides a measure of the intensity of wide-angle scattering that is due to the underlying mica substrate alone. The majority of the ROI exhibits intensity marked as ‘tissue’ in this plot, with neither particularly high or particularly low intensity. Regions of low intensity correspond to disrupted tissue with a high density of voids (and corresponding high scattering exponent). The intensity of scattering from these regions, although relatively low, is significantly higher than observed in the lumen of the blood vessel, thereby demonstrating that the tissue remains intact in spite of the relatively high abundance of sub-micron sized voids. Lesions and other high density tissue features exhibit high scattering intensity in the WAXS regime.

### 3.3 Comparison of scanning x-ray microdiffraction with images of silver-stained tissue

After x-ray exposure, the thin sections were detached from the underlying mica substrate, transferred to glass slides and subject to silver staining. In most cases, bleaching due to x-ray exposure facilitated accurate registration of images of the silver-stained sections with corresponding maps of x-ray scattering attributes. Some sections underwent gross distortion during staining, precluding accurate registration. However, for those sections that were not distorted during silver staining, comparison of the distribution of x-ray attributes with features in the images of the silver staining was highly informative. Most dramatically, the locations of pockets of high scattering exponent (large abundance of scattering voids) proved to correspond closely to locations where tissue damage (rips, tears, holes) occurred during silver staining (after x-ray exposure).

**Figure 3:**
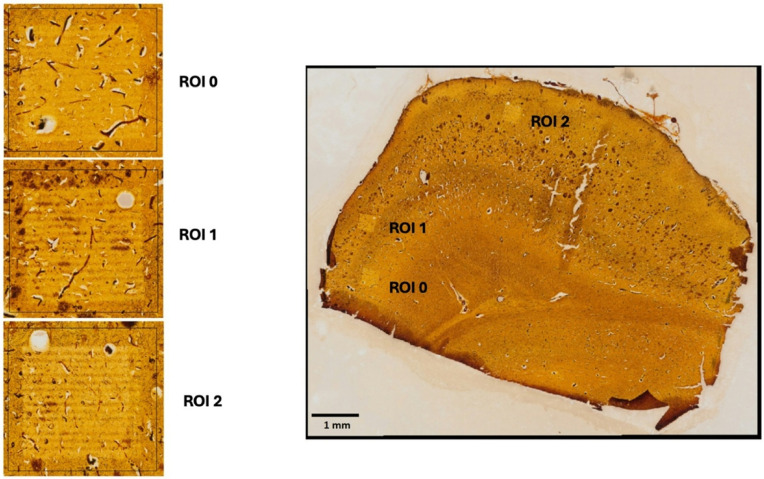
Tissue section from the entorhinal cortex of a subject staged at Braak VI and silver stained after exposure to scanning x-ray microdiffraction. Three 300×300 μm^2^ ROIs were scanned with a 5 mm x-ray beam, lowering the affinity of the tissue for silver ions and marking the regions exposed. Close examination of the ROIs (insets) indicates that ROI1 and ROI0 have lower burden of neuropil deposits compared to ROI2 (although not lower overall density of staining).

**Figure 4:**
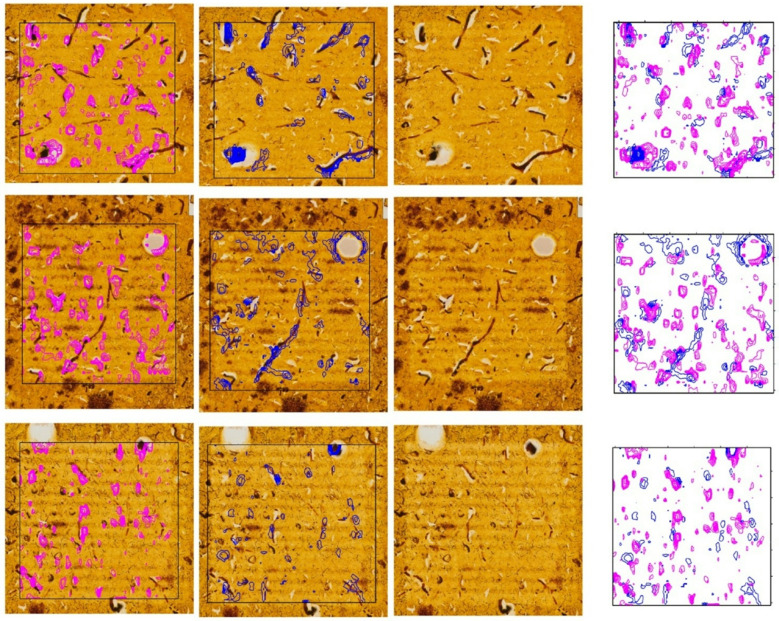
Comparison of images of three ROIs from a silver-stained tissue section (entorhinal cortex, Braak VI) with corresponding heat maps of WAXS intensity (blue contours) and scattering exponent (pink contours). Dark-staining features in the image of silver-stained tissue are usually correlated with positions of high WAXS intensity. Small rips, tears or holes in the tissue, generated during silver staining after x-ray exposure often correlate with regions of greatest scattering exponent – which correspond to regions with abundant voids – voids which apparently imbued the tissue with a local vulnerability to physiochemical treatment during staining. Left hand column is superposition of distribution of scattering exponent and silver stained image. Middle column is superposition of distribution of wide-angle intensity on an image of the silver stained section. Right hand column is the image of the silver-stained section with no overlay - repeated for clarity. In the far right column are the corresponding distribution of wide angle scattering (blue) and scattering exponent (pink).

The use of silver staining to visualize cellular architecture in sections that had been previously examined with scanning x-ray microdiffraction was made easier due to bleaching of the tissue by the x-rays. X-ray exposure ejects electrons from the surface of the tissue lowering its affinity for the silver ions used for staining, marking the regions that have been exposed to x-rays and facilitating registration of micrographs of silver-stained tissue with heat maps constructed from the x-ray data. Figure 3 shows an image of a silver-stained section from the entorhinal cortex of a Braak VI subject of which three 300×300 μm^2^ ROIs were scanned. Enlargements of the three ROIs are shown as insets in Fig. 3 and correspond to the heat maps of WAXS intensity and scattering exponent in Fig. 4.

Virtually all dark-staining features in the image of the silver-stained section are also apparent in the contour map of WAXS intensity (blue contours). This includes features that have been identified as vascular walls, including, for instance, in ROI1, a small penetrating blood vessel in the upper right and a long feature tangential to the section and representing another blood vessel running through the lower middle portion of the ROI. The image of the silver-stained section exhibits small rips and tears that were generated during the manipulation required for silver staining of the section. These breaks in the tissue were not present at the time of x-ray exposure as can be seen from the transverse view of WAXS intensity from the same ROI in Fig. 2c. Nevertheless, these rips correspond closely to the regions with highest scattering exponent - in other words, those regions with the largest number of voids. Apparently, the presence of voids makes the tissue vulnerable to damage during silver staining, leading to the mechanical disruptions apparent in images of the silver-stained tissue.

### 3.4 Correlation of protein accumulation with scattering exponent

**Figure 5:**
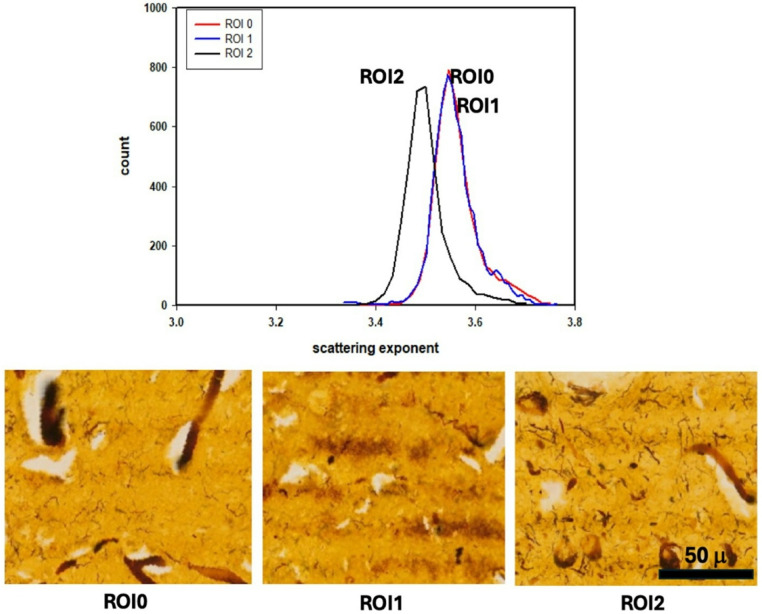
Images of different regions of interest (ROIs) in a single silver-stained section reveal noticeable differences in neuropil density. (top) Histograms of the scattering exponent in three ROIs from the same tissue section. The distribution of scattering exponents observed for ROI 2 is distinctly shifted relative to ROIs 0 and 1. (bottom). Images of the silver-stained ROIs stained after x-ray exposure. Close examination indicates increased local neuropil and pathological density in ROI 2.

Histograms of the scattering exponent in the three ROIs of Fig. 4, indicate that ROI2 has, on average, significantly lower scattering exponents than observed in ROI0 or ROI1 (Fig. 5; top). Close examination of the images of silver-stained sections reveals an abundance of neuropils and other diffuse deposits within the tissue as seen in Fig. 5 (bottom panels). These features are too small to be detected individually with a 5 μm x-ray beam, but the overall impact of their presence causes the observed shift in the value of the scattering exponent. Close examination (Fig. 5 bottom panels) of the images of silver-stained ROIs indicates that ROI 0 and ROI 1 have a lower burden of diffuse deposits than ROI 2. This is reflected in a larger average scattering exponent in these ROIs. Apparently, the lower density of diffuse protein deposits in ROI 0 and ROI 1 allowed the formation of a greater abundance of voids during tissue preparation, which was reflected in an increase in average scattering exponent as seen in the histograms in Fig. 5. The histogram for ROI 2, in which protein deposits are densest, is shifted significantly to the left compared to those for the other ROIs, indicating a smaller average size of voids.

### 3.5 Distribution of pockets with high abundance of sub-micron voids

**Figure 6:**
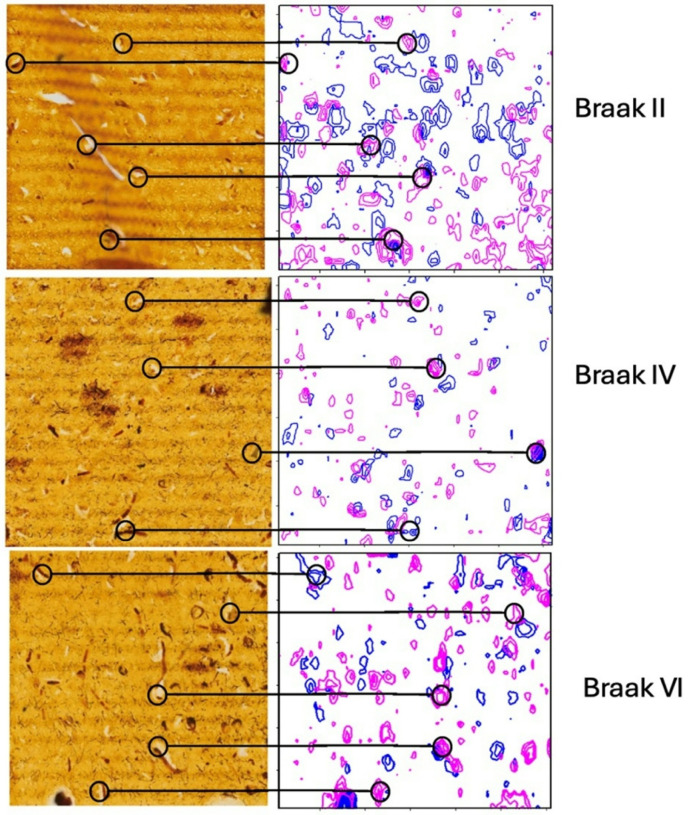
Illustration of the correspondence of peaks in the scattering exponent distribution (indicating regions with an abundance of large voids) with locations of tissue damage observed in the silver-stained section. Only a few representative locations are marked, but close examination will reveal many more places where the two measures coincide.

Figure 6 provides additional demonstration of the correspondence of features in the silver-stained images and the x-ray heat maps as exhibited by the distribution of scattering exponent for an ROI from the EC of three subjects staged at Braak II, Braak IV and Braak VI. Selected peaks in the distribution of scattering exponent are marked along with the corresponding position of tissue damage apparent in the image of the section after silver staining.

**Figure 7:**
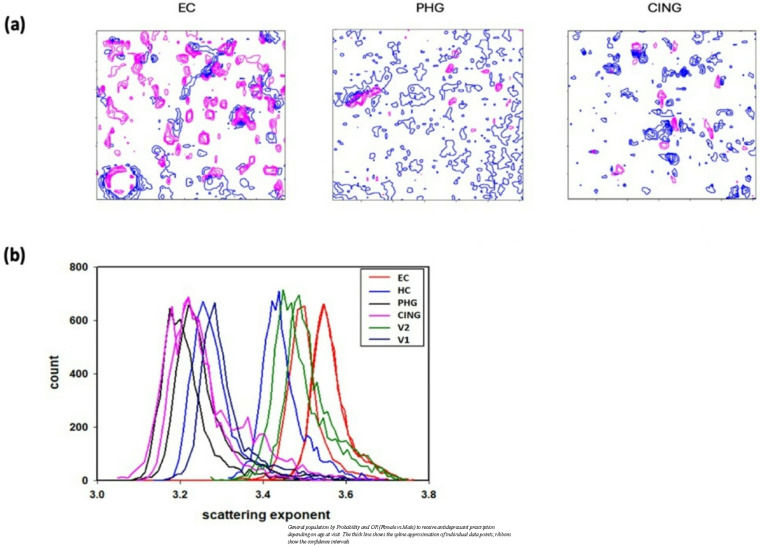
Attributes of Braak VI subject **(a)** Distribution of voids and high-density features ROIs from the entorhinal cortex (EC), the parahippocampal gyrus (PHG) and anterior cingulate cortex (CING) from the same Braak VI subject. All contour maps are 300×300 mm^2^. **(b)** Histograms of scattering exponent for 11 ROIs from six regions of the brain of a Braak VI subject.

Figure 7a displays the distribution of voids and high-density features in ROIs from the entorhinal cortex (EC), the parahippocampal gyrus (PHG) and anterior cingulate cortex (CING) from the same Braak VI subject. In this selection, the regions of large voids are far more common in the EC than in the PHG or CING. Since the progression of neurodegenerative processes also moves from EC to PHG and then to CING, this observation suggests that the level of neurodegeneration within a tissue might contribute to the formation of these vulnerable regions. Nevertheless, variation within individual tissue sections appears at least as great as among brain regions. This could reflect a complex trajectory of neurodegeneration, or the contribution of anatomical features or other processes to the breakdown of tissue.

In Fig. 7b, histograms for the scattering exponent for 11 ROIs in six regions of the brain of a Braak VI subject are plotted. Data were collected for three ROIs in the EC, two in the HC, PHG, CING and V2 (associative visual cortex) and one in the V1 (primary visual cortex). In the PHG, CING and V2, the distribution of scattering exponent was roughly the same for the two ROIs, indicating relatively uniform distribution of diffuse protein accumulation. For the HC, which is anatomically diverse, the two ROIs exhibited rather different degrees of protein accumulation. For the EC, as detailed above, two of the ROIs had virtually identical distribution of protein deposition, while one had significantly more (as indicated by a lower density of voids).

### 3.6 Mapping voids along the Braak-tau trajectory

Comparison of histograms of scattering exponent determined for different ROIs in different tissue sections is made possible by its invariance to tissue thickness or changes in the intensity of the incident x-ray beam. This makes it a valuable parameter for comparing properties of tissue in different regions. Conversely, comparison of histograms of WAXS intensity from different ROIs is intrinsically less certain because intensity might vary due to small variations in experimental parameters that may be difficult to adequately control (e.g. thickness of tissue section being scanned). This may lead to apparent differences among ROIs that do not reflect material properties. Consequently, we focus on scattering exponent in region-to-region comparisons.

The scattering exponent obtained from linear fitting of the observed small-angle intensities can be used to estimate the average radius of voids in a single scattering volume using an empirical relationship (Nepal et al., 2024). The average void radius in a single scattering volume is proportional to the scattering exponent *p* observed in scattering from that volume. A larger number or size of voids will give rise to a higher scattering exponent.

**Figure 8:**
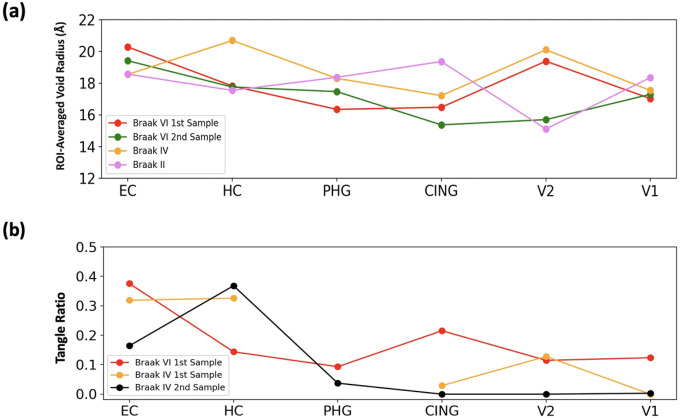
(a) ROI-averaged void radius and (b) Tangle ratios mapped across brain regions for samples from subjects at three different Braak stages. Tangle ratio was calculated from immuno-tau-stained images of serial sections prepared in coordination with the unstained sections analyzed by scanning x-ray microdiffraction.

Thus, we chose the ROI-averaged void radius as a measure to investigate the variation of tissue properties across regions of the brain. Within an ROI, the void radii calculated for each scattering pattern was distributed in a near-Gaussian distribution as seen in the distribution of scattering exponents plotted in Figs. 5 and 7. Figure 8a is a mapping of the ROI-averaged void radius calculated from those distributions as a function of brain region (subjects with partial data omitted). Each data point in this plot represents the average of at least 3600 scattering patterns. Because of the heterogeneity of tissue within each region, these numbers do not necessarily reflect an overall property of the tissue. The greatest contributor to uncertainty in this plot is the choice of ROI. Consequently, no statistically significant trend could be derived from these data.

Tau pathology in the corresponding tissue was tracked using tangle segmentation for images corresponding to different Braak stages as performed using a pixel classifier. Tangle counts were validated through visual inspection. An immediate advantage of using tangle ratio (expressed here as a ratio between the number of pixels identified as tangle and the total number of pixels analyzed) is that they provide a more quantitative measure than Braak stage classification alone. Variation in the tangle ratio (proportion of pixels containing tangles) exhibited a slow decrease as a function of distance along the Braak-Tau trajectory as seen in Fig. 8b.

## Discussion

In this paper, we have described a quantitative analysis of macromolecular density and tissue loss within human brain tissue in Alzheimer’s disease based on small- and wide- angle x-ray scattering. Interpretation of these data has been greatly enhanced by the correlation of this data with images of silver-stained tissue. At the scale of tens of microns, the mapping of regions of high WAXS intensity – which correlates with high protein density - has provided the locations of high-density features including vascular walls and pathological protein deposits. These correlate well with darkly staining features in images of silver-stained tissue. More surprisingly, the mapping of scattering exponent provided information on the location of pockets – typically 20–50 μm in diameter - of greater porosity as indicated by greater abundance of sub-micron sized voids. Correlating the locations of these pockets with features in images of silver-stained sections surprisingly demonstrated that they have lower than average physical integrity. Their locations correlate remarkably well with the locations of rips or tears that were generated after x-ray exposure by the preparation of the sections for silver-staining. The origin of these diaphanous pockets is unknown. They most likely formed during sectioning and tissue processing. But what they might tell us about the properties of tissue prior to histological processing in unclear.

While we have focused on high-density features and low-density pockets, the properties of the (majority of) tissue located between these features are worthy of note. High-density features described here correlate with features readily observed by standard histological methods and low-density pockets provide a novel ‘positive control’ that both identifies regions of low physical integrity and defines the range of variation occurring in tissue. But scattering provides completely new observations of the ‘regions in between’ which are not readily characterized by conventional tools.

In virtually all the ROIs we studied, the intensity of wide-angle scattering is distributed in a near-Gaussian fashion as is the distribution of scattering exponent (see Figs. 5 and 7). The breadth of these Gaussian distributions appears relatively constant, perhaps reflecting a preservation of some fundamental attributes of tissue architecture across brain regions and stages of disease. By contrast, the average of these distributions varies widely, not just among different regions of the brain, but also within a single tissue section (see Fig. 5). Since SAXS data derives largely from the presence of voids, this variation informs us as to the variation in the micro-porosity of tissue. Comparing variation in scattering exponent with variations in the appearance of tissue that has been silver stained indicates that the density of diffuse protein accumulation – as exemplified by the presence of an abundance of neuropils and other amorphous protein structures – correlates inversely with the scattering exponent. Neutropils have dimensions far smaller than tangles or plaques, but their deposition may generate an overall increase in macromolecular density throughout the diseased tissue. ROIs with greater average scattering exponent (more voids) exhibit far less diffuse protein accumulation. This can be seen in Fig. 5 which contains a detailed analysis of three ROIs from a single section of EC in the brain of an individual with advanced disease (Braak VI). Not surprisingly, higher levels of protein accumulation hinder the formation of voids. While the correlation of scattering exponent to diffuse protein accumulation can be understood, the origin of the near invariance of the breadth of the scattering exponent distribution remains obscure.

It is tempting to hypothesize that during disease progression, the amount of diffuse protein deposition initially increases, but at the point where cell death becomes common, clearance of cellular debris might decrease overall protein density level. This is consistent with the observation that, in some cases, the abundance of pockets of high tissue porosity decreases as a function of distance along the Braak-Tau trajectory (as in Fig. 7). However, within the limited set of data collected here (~ 56 ROIs and ~ 200,000 + scattering patterns) variation within a single tissue section was nearly as great as that observed across all 6 of the brains examined. An impractically large data set would be required to provide statistically significant evidence of systematic variation in the density of protein accumulation during disease progression. See Fig. 8.

As AD progresses through Braak stages, the composition, orientation, and internal organization of tissue constituents may change significantly (Braak and Braak, 1991; Thal et. al. 2002). Relative tangle counts generally increase with Braak stages; and higher tangle burdens are often linked to increased neurodegeneration. Higher tangle counts are expected at earlier positions in the Braak-Tau trajectory as suggested by the data in Fig. 8b. Neurodegeneration that leads to cell death will ultimately be expected to generate pockets of low tissue density which may foster void formation. On a macro scale, these pockets may contribute to the gross atrophy observed in the brains of Alzheimer’s subjects (Tarasoff-Conway et.al. 2015).

The combination of x-ray scanning and optical images of silver-stained materials indicates that scattering voids are a feature of these tissue sections that is not completely captured by standard histology. The mechanisms underlying the origin, formation, and prevalence of these scattering voids remain to be fully understood. Three possible mechanisms can be considered: (a) tissue density is inherently low around regions that have undergone neurodegeneration, leading to the formation of voids during sample preparation or dehydration; (b) formation of low-density regions through localized stretching of tissue during sample preparation; (c) void formation may be related to the extraction of tissue lipids during sample processing, especially in regions where lipids are highly concentrated. Regions that eventually appear as voids may have previously contained higher lipid levels than their surroundings, making the voids particularly conspicuous after processing. Loss of cellular material in localized pockets may result in increased porosity (Karunasena et. al. 2015) and higher number of voids. Regions with the highest prevalence of voids may be associated with cellular death and clearance. Other changes in tissue environment induced by sample preparation have been reported (Quester and Schroder, 1997), but their impact on void distribution remains to be investigated. A complete understanding of what the scattering voids represent is elusive. Nevertheless, their characterization provides a novel approach to studies of the changes that occur within a brain.

The observation of a linear log(I)-log(q) dependence in the small-angle regime is sufficiently universal that one expects it to arise from a fundamental property of materials. The extensive analysis of scattering on the basis of fractal-like molecular architecture arose from this expectation (Bak 2013). The data is consistent with the presence of fractal structure. Nevertheless, two properties of the tissues investigated here preclude that possibility. First, the molecular structures making up the tissue are well understood and are not capable of interacting in a way as to construct fractal-like architectures. Second, the inverse correlation between SAXS intensity and WAXS intensity points to voids – sub-micron sized air bubbles – as the major contributor to SAXS data. Accepting that, we still need to account for the fact that in scattering from all of these tissues the very small-angle intensity exhibits a linear log-log dependence on q. This, again suggests a universal principle underlying the observations. The linear log-log dependence cannot be explained on the basis of a homogeneous population of void sizes (Nepal et al., 2024). Although there may be many distributions of void sizes that could generate the observed, we demonstrated that a scale-free distribution (in which the number, N, of voids of radius, r were distributed according to N(r) ~ r^−a^ where ‘a’ need not be an integer) of void sizes was consistent with observations. If this distribution is universal, as it seems to be, it would suggest a universal mechanism for growth and coalescence of voids in which larger voids grow at greater rates.

## Data Availability

The datasets used to support the conclusions of this study will be available from corresponding authors, without reservation.
